# Associations of the Total Cholesterol/high-density Lipoprotein Cholesterol Ratio with Outcomes in Lupus Nephritis

**DOI:** 10.7150/ijms.106393

**Published:** 2025-04-28

**Authors:** Xiaolei Shi, Yaoyao Tang, Wang Xiang, Jianwen Yu, Xin Wang, Hongjian Ye, Zhong Zhong, Haishan Wu, Ruihan Tang, Xi Xia, Wei Chen

**Affiliations:** 1 Department of Nephrology, The First Affiliated Hospital of Sun Yat-Sen University, People's Republic of China.; 2 NHC Key Laboratory of Clinical Nephrology (Sun Yat-Sen University) and Guangdong Provincial Key Laboratory of Nephrology, People's Republic of China.

**Keywords:** Lupus nephritis, Total cholesterol/high-density lipoprotein cholesterol (TC/HDL-C) ratio, Prognosis, All-cause mortality.

## Abstract

**Objectives:** Dyslipidemia is common in lupus nephritis (LN). However, the relationship between the total cholesterol/high-density lipoprotein cholesterol (TC/HDL-C) ratio and LN remains unclear. This study was designed to investigate the association between the TC/HDL-C ratio and LN.

**Method:** This study included individuals diagnosed with LN between January 1, 1996 and December 31, 2018. Split by the optimal cutoff TC/HDL-C ratio value of the primary outcome, patients were divided into lower (<6.71) and higher (≥6.71) TC/HDL-C ratio groups. Multivariate Cox regression analysis and subgroup analyses were carried out to confirm the connection of the TC/HDL-C ratio with the adverse clinical outcomes in LN.

**Results:** A total of 818 patients with LN were followed up for a median of ten years and 129 (15.77%) experienced all-cause death and 119 (14.55%) reached adverse renal events. Kaplan-Meier survival analyses demonstrated that patients exhibited a higher TC/HDL-C ratio were more susceptible to all-cause death (P=0.003) and adverse renal outcomes (P=0.001) in LN. After adjustments, a higher TC/HDL-C ratio still exhibited significant correlations with all-cause death [hazard ratio (HR):1.51, 95% confidence interval (CI): 1.03-2.23; P=0.036] and adverse renal outcomes in LN patients [HR: 1.57, 95%CI: 1.05-2.36; P=0.028]. Further subgroup analyses revealed that LN patients who were male, younger than 40 years old or with estimated glomerular filtration rate under 60 ml/min/1.73m2 seemed to be more susceptible to adverse clinical outcomes (P<0.05).

**Conclusions:** An elevated TC/HDL-C ratio exhibited significant associations with poor prognosis in LN. Patients with LN may benefit from further TC/HDL-C studies.

## Introduction

Lupus nephritis (LN) is prevalent in approximately 50-60% of patients with systemic lupus erythematosus (SLE) and this serious condition causes distress and severe socioeconomic hardship for these patients 1, 2. Patients with LN experience a substantial risk of serious renal complications and increased all-cause mortality 3, 4. However, due to the heterogeneity of LN, prognostic assessment using non-invasive tools remains challenging.

Dyslipidemia, often defined as aberrant levels of the fasting total cholesterol (TC), triglycerides (TG), low-density lipoprotein cholesterol (LDL-C), or high-density lipoprotein cholesterol (HDL-C), is frequently observed among LN patients 5, 6. Dyslipidemia may induce adverse clinical outcomes by promoting the production of inflammatory cytokines and cardiovascular events 7, 8. Our previous findings revealed that low HDL-C levels increased the incidence of all-cause death, while high HDL-C levels increased the probability of end-stage renal disease (ESRD) in LN patients 9. These results raised a question that when considering lipid-lowering therapy, targeting HDL-C alone may fail to reduce the likelihood of both all-cause death and ESRD. Therefore, identifying a comprehensive index with improved predictive value is imperative.

The TC/HDL-C ratio was recently validated as an indicator for adverse clinical outcomes in multiple diseases and contributes better predictive value than TC or HDL-C 10-13. Increased TC and decreased HDL-C are connected to the increase possibility of developing chronic kidney disease (CKD) 14-16. Additionally, the TC/HDL-C ratio is also independently related to high-risk of CKD progression 14. Studies have shown that compared to SLE patients without kidney involvement, the TC was much higher and showed a significantly positive correlation with 24h urine protein in LN patients 8. Combining this evidence with our previous results, we speculated that the TC/HDL-C ratio might be related to LN prognosis.

To address this knowledge gap, this study focuses on the independent forecast capability of the TC/HDL-C ratio to predict poor clinical outcomes among LN populations, potentially offering valuable insights into therapeutic strategies and prognosis in this distinct cohort.

## Methods

### Study design and enrolled population

This is an observational study conducted based on a single center, ambispective cohort study (High Quality Evidence of Guangzhou Lupus Nephritis Cohort [HOPE Cohort], NCT06682507) in China. Patients who received a diagnosis of LN in the period between 1 January 1996 and 31 December 2018 were enrolled as participants. Diagnostic criteria followed the American College of Rheumatology 1997 revised criteria for SLE and the RPS 2003 pathological classification criteria for LN 17, 18. The patients before 1997 were diagnosed by reviewing medical records. Exclusion criteria: (1) age under 14 years old, (2) baseline diagnosis of ESRD, (3) diagnosis of a malignant tumor, (4) drug-induced lupus erythematosus, (5) absence of serum fasting lipid data, (6) renal biopsy specimens with fewer than 10 glomeruli, and (7) follow-up <6 months or absence of follow-up data. All patients were followed up until they reached the endpoints of the study or until September 30, 2023. This study was approved by the Human Ethics Committee of Sun Yat-sen University (No. 2016-215).

### Data collection

Baseline demographic information, clinical data, and biochemical parameters were collected from each patient upon their initial hospital admission for renal biopsy. Renal pathology data were collected from the first renal biopsy of LN diagnosis. Blood samples for hemoglobin, fasting blood glucose, serum creatinine, urine acid, serum albumin, lipid profiles, antibodies, complement, erythrocyte sedimentation rate (ESR) were obtained after overnight fasting. The estimated glomerular filtration rate (eGFR) was derived from the CKD-EPI equation formula 19. The SLE disease activity index (SLEDAI) was evaluated utilizing the SLEDAI-2K scoring system 20.

### Study outcomes and definitions

The primary outcome was defined as all-cause mortality. The secondary outcomes were defined as adverse renal outcomes, including serum creatinine doubled and ESRD (eGFR < 15ml/min/1.73m^2^ or maintenance dialysis, or kidney transplantation). Hypertension was defined as repeated systolic blood pressure ≥140 mmHg and/or diastolic blood pressure ≥90 mmHg. Nephrotic syndrome was defined as nephrotic proteinuria (>3.5 g/24h) and hypoalbuminemia (serum albumin <30 g/L). Pathological classifications were defined as proliferative (including Class III, Class IV, Class III/IV plus V, and Class VI) and non-proliferative LN (Class I, Class II, and purely Class V) according to our previous research 21.

### Statistical analysis

The optimal ratio of TC to HDL-C for predicting the primary outcome was 6.71 determined according to the receiver operating characteristic (ROC) curve analysis (Table S1). Based on this optimal cutoff value, patients were divided into the lower (<6.71) and higher (≥6.71) TC/HDL-C groups, respectively. The Kruskal-Wills test or the chi-square test were used to compare the differences among the groups. Categorical variables were presented as numbers and percentages. Continuous variables were described as mean ± standard deviation or median with interquartile range.

Clinical outcomes were evaluating using the Kaplan-Meier(K-M) method. Three-knot restricted cubic spline (RCS) analyses were conducted to assess the non-linear and exposure-dose relationships between the continuous TC/HDL-C ratio and adverse clinical outcomes. Unadjusted and adjusted multivariate Cox regression models have been performed to assess the relationships between poor clinical outcomes and TC/HDL-C. We conducted three models based on the adjustment of different indicators for each analysis. In the fully adjusted model (Model 3), variables considered to be potential confounder for adverse prognosis of LN were adjusted, including gender, age, weight, glucocorticoids, immunosuppressants, lipid-lowing treatment, eGFR, nephrotic syndrome, IgG, ESR, activity index, and chronicity index. Subgroup analyses stratified by gender, age, hypertension, nephrotic syndrome, eGFR categories and pathological classification were carried out in the Model 3 to confirm the consistent prognostic influence of TC/HDL-C on clinical outcomes. A *P* value below 0.05 was regarded as statistical significance. Packages from R (4.3.2) were utilized for all statistical analyses in this study.

## Results

### Baseline clinical features

**Figure 1:**
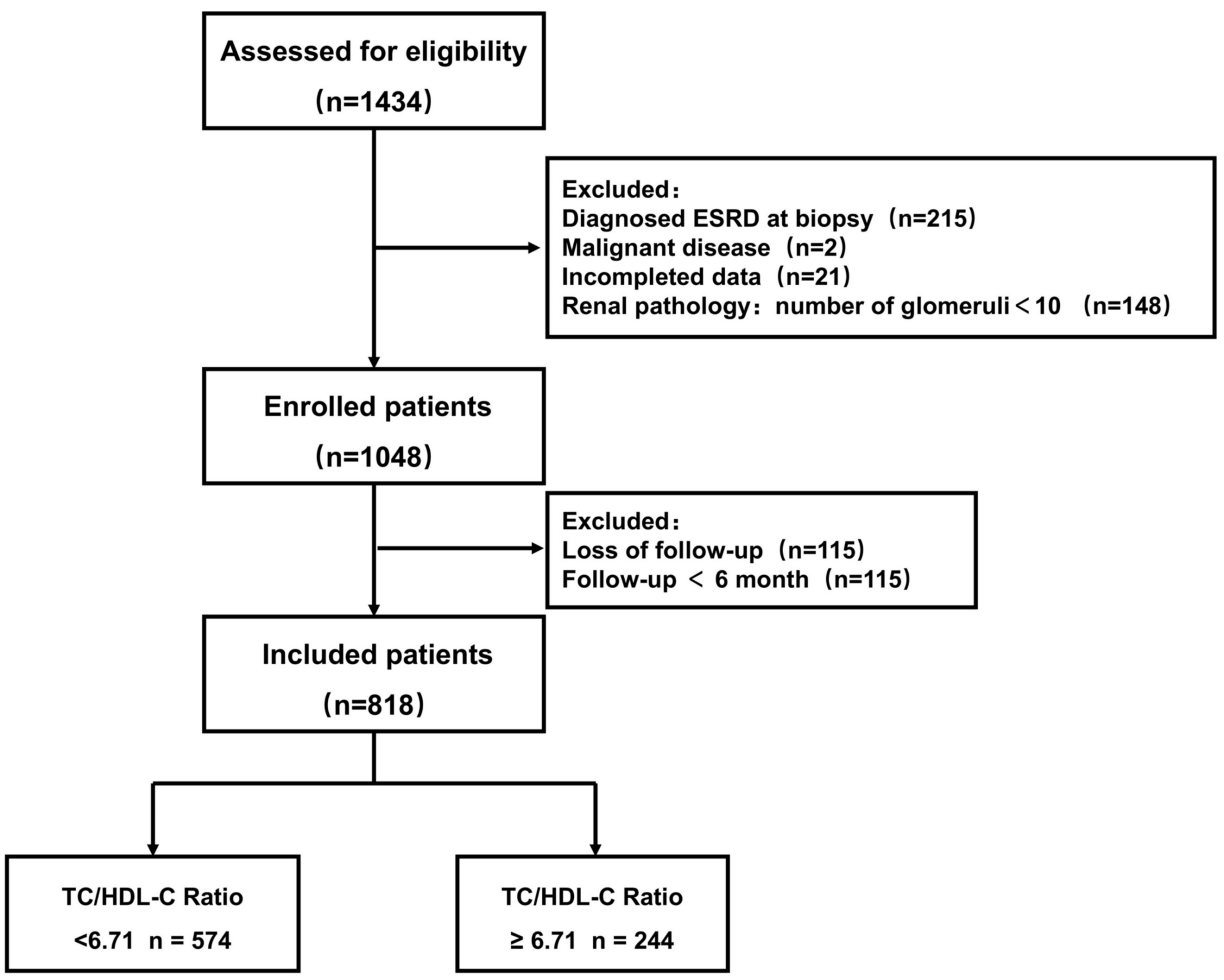
Study flowchart.

**Figure 2:**
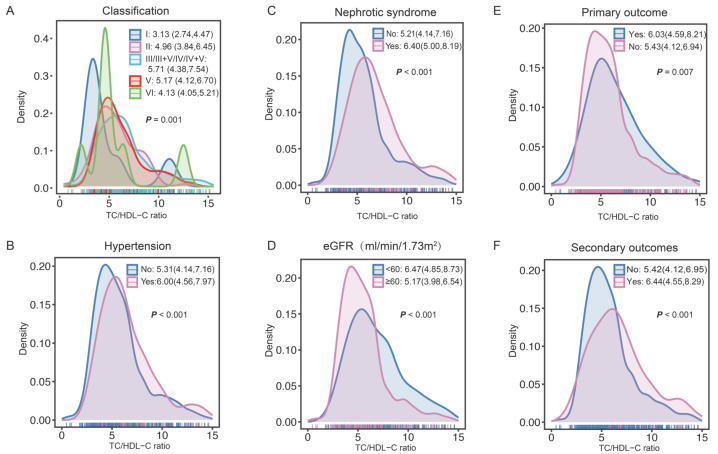
** The TC/HDL-C ratio distributions at baseline in the subgroups. A** The TC/HDL-C ratio distributions in the subgroups of pathological classification. **B** The TC/HDL-C ratio distributions in the subgroups of hypertension. **C** The TC/HDL-C ratio distributions in the subgroups of nephrotic syndrome. **D** The TC/HDL-C ratio distributions in the subgroups of eGFR. **E-F** The TC/HDL-C ratio distributions in the subgroups of primary outcome and secondary outcomes.

After screened by the inclusion and exclusion criteria, overall, 818 patients diagnosed with LN were enrolled (Figure 1). Overall, the mean age was 27 years. Among these patients, 83.25% were female, 33.86% patients with hypertension, and 27.51% manifested as nephrotic syndrome. Table 1 presents the differences of clinical and pathological characteristics at baseline between the lower and higher TC/HDL-C ratio groups. Significantly increased percentages of hypertension, nephrotic syndrome, and lipid-lower treatment were observed in LN patients with a higher TC/HDL-C ratio (All *P<*0.05). Significant differences were observed in the distributions of baseline TC/HDL-C ratio in terms of pathological classification, nephrotic syndrome, hypertension, renal function, primary outcome and secondary outcomes. (All *P<*0.05) (Figure 2).

The 24h proteinuria, SLEDAI scores, ESR levels and percentage of positive antinuclear antibody were statistically increased in the higher TC/HDL-C ratio group (All *P<*0.05). However, the levels of hemoglobin, eGFR, serum albumin, IgG and serum complement C3 were significantly lower in LN patients with higher TC/HLD-C ratio (All *P*<0.001). In terms of pathological characteristics, the higher TC/HDL-C ratio group showed statistically higher levels of activity index and chronicity index, much higher proportions of crescents, interstitial fibrosis, platinum loop, microthrombus, and glomerular leukocyte infiltration (All *P<*0.05).

### The TC/HDL-C ratio and prognosis of LN

During a median follow-up of ten years, the occurrence of all-cause death and adverse renal endpoints were 129 (15.77%) and 119 (14.55%) patients, respectively. The main causes of death in this study were infection (35/129, 27.13%), renal failure (21/129, 16.28%) and cardiovascular or cerebrovascular events (26/129, 20.16%). As shown in the Table S2, the mortality of the higher TC/HDL-C ratio group (22.54%) was significantly higher than that of lower TC/HDL-C ratio group (12.89%) (*P*<0.001).

**Figure 3:**
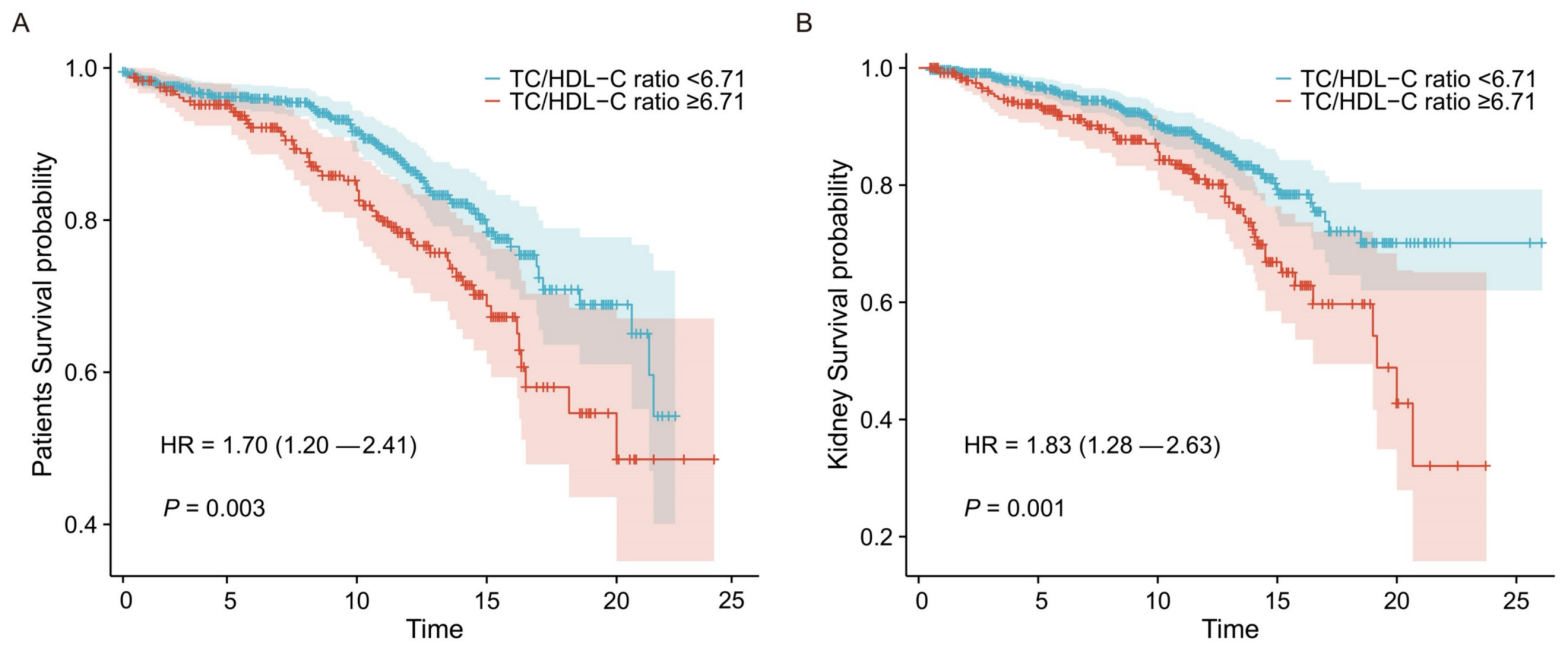
** Associations between the TC/HDL-C ratio groups and clinical outcomes in LN.** Kaplan-Meier survival curves for all-cause mortality (A) and adverse renal outcomes (B) in the TC/HDL-C ratio groups of LN patients.

The K-M survival analyses of the two groups for clinical outcomes are presented in Figure 3. Statistical significance was observed in the groups of lower and higher TC/HDL-C ratio, with both worse patient and renal survival (*P*=0.003 and *P*=0.001, respectively) in the higher TC/HDL-C group.

To provide further evidence for the better predictive ability of the TC/HDL-C ratio compared to individual parameters, we also investigated the impact of TC, HDL-C or LDL-C levels on overall mortality and renal survival (Figure S2). Patients were also stratified by the optimal cut off value predicting the primary outcome according to the ROC curves (Table S1). No significant difference of all-cause mortality and renal survival between lower and higher TC groups (All *P*>0.05). Renal survival rates were lower in the higher LDL-C groups compared to the lower LDL-C groups (*P*=0.037). And consistent with our previous study, lower HDL-C levels have a connection with higher likelihood of all-cause death in LN (*P*<0.001).

Further multivariate cox regression models were performed to assess the adverse clinical outcomes by the continuous TC/HDL-C ratio and TC/HDL-C categories (Table 2). The reference group was defined as the lower TC/HLD-C ratio group. After adjusting for gender, age, weight, glucocorticoids, immunosuppressants, lipid-lowing treatment, eGFR, nephrotic syndrome, IgG, ESR, activity index, and chronicity index in Model 3, in comparison to the reference group, the hazard ratios (HRs) for all-cause death and adverse renal outcomes in the higher TC/HDL-C ratio group were 1.51 [95% confidence interval (CI): 1.03-2.23; *P*=0.036] and 1.57 [95%CI: 1.05-2.36; *P*=0.028], respectively. The prognostic value of continuous TC/HDL-C ratio for all-cause mortality [HR:1.07, 95%CI: 1.02-1.12; *P*=0.010] and adverse renal outcomes [HR:1.06, 95%CI: 1.01-1.11; *P*=0.015] in LN was also detected in Model 3.

**Figure 4:**
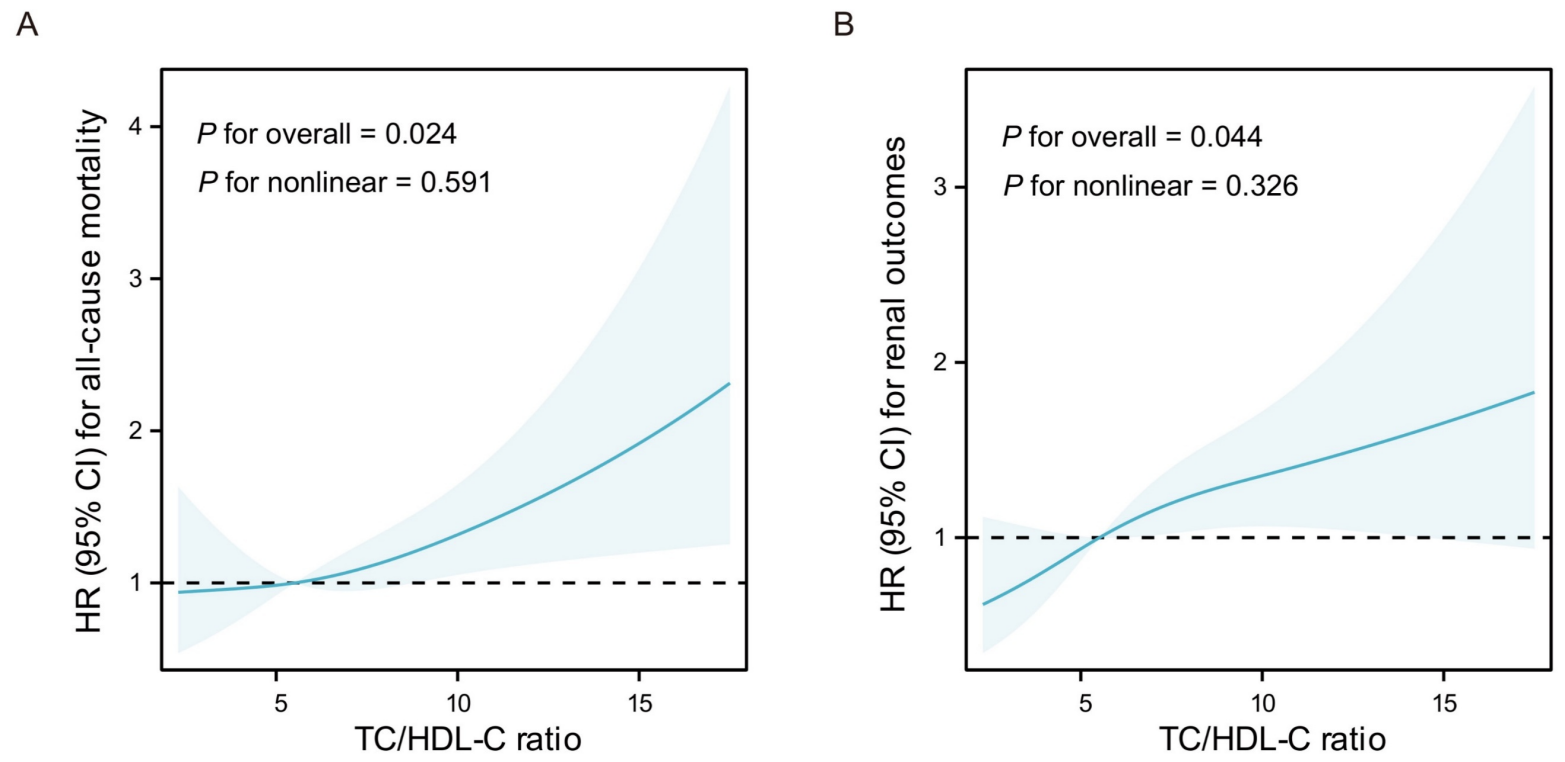
** Results of adjusted three-knot RCS models. A** Association between the TC/HDL-C ratio and all-cause mortality in LN. **B** Association between the TC/HDL-C ratio and adverse renal outcomes in LN. The solid lines indicate HR, shadow shapes indicate 95% CI. Models were adjusted for gender, age, smoking, weight, eGFR, proteinuria, Complement C4, IgG, ESR, pathological classification, activity index, chronicity index, and lipid-lowing treatment. HR: Hazard Ratio, CI: Confidence Interval.

According to the results of three-knot RCS models, liner correlations have been observed between all-cause mortality or adverse renal outcomes and the continuous TC/HDL-C ratio in LN (*P* for nonlinear = 0.591 and 0.326, respectively). Additionally, the liner dose-response relationships were found between the TC/HDL-C ratio and all-cause mortality (*P*=0.024) as well as adverse renal outcomes (*P*=0.044) in Model 3 (Figure 4).

### Subgroup analyses

**Figure 5:**
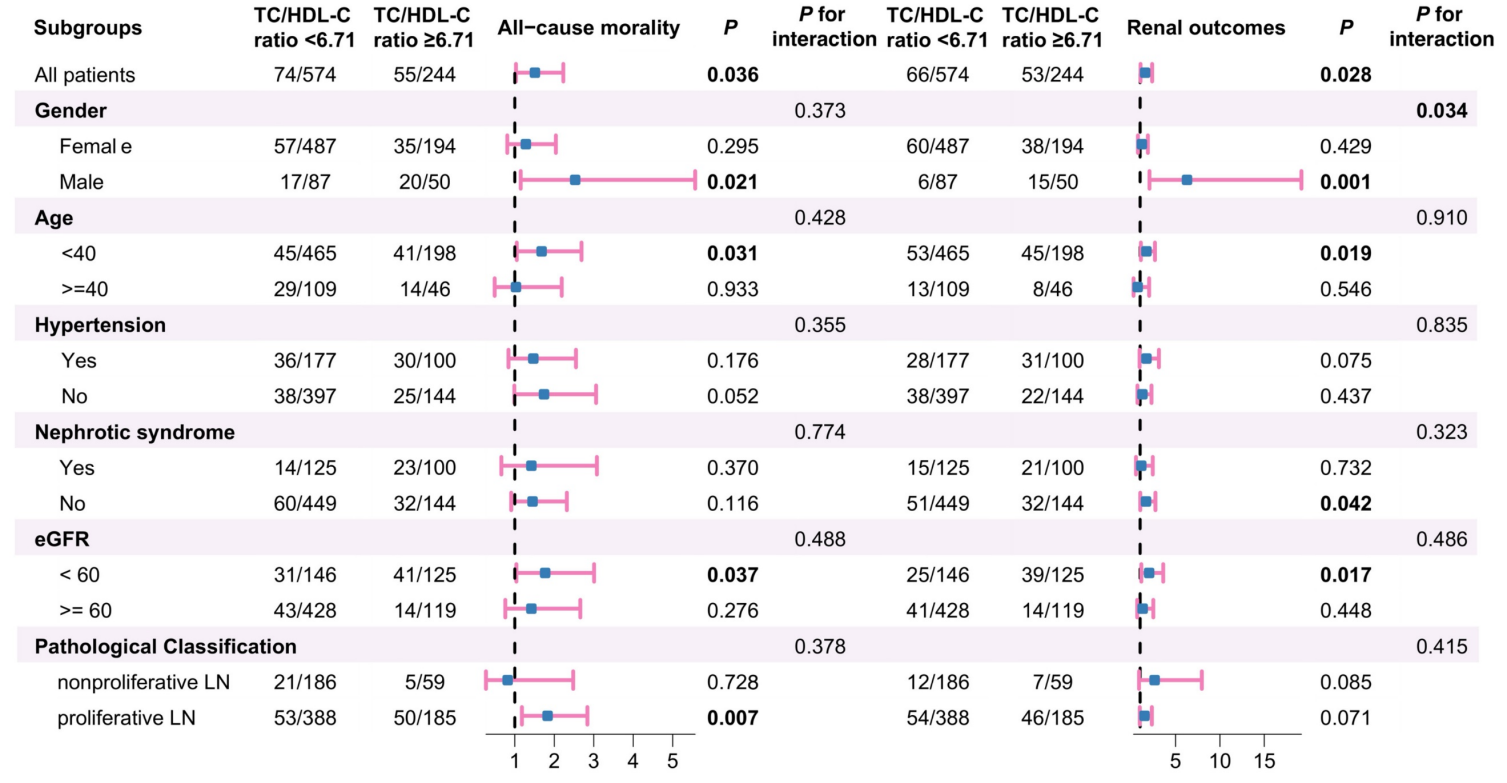
Subgroup analyses for the association between the TC/HDL-C ratio and clinical outcomes of LN.

Next, subgroup analyses were carried out based on gender, age, hypertension, nephrotic syndrome, eGFR categories and pathological classification to examine the impact of population stratification on the relationship between TC/HDL-C subgroups and patient or renal survival in LN (Figure 5). The analyses revealed that LN patients with higher TC/HDL-C ratio who were male, aged under 40 years old, with eGFR under 60 ml/min/1.73m^2^ or without nephrotic syndrome showed a statistically increased likelihood of adverse renal outcomes (All *P*<0.05). Besides, LN patients with higher TC/HDL-C ratio who were male, aged under 40 years old, manifested as eGFR < 60 ml/min/1.73m^2^ or proliferative pathological injuries were more susceptible to overall mortality (*P*<0.05). Further interaction tests indicated that gender may influence the association between RC and poor renal outcomes in LN (*P* for interaction=0.034).

## Discussion

Based on an extensive literature review, this cohort study conducted in Southeast China represents the first and largest investigation to date exploring the evidence linking the TC/HDL-C ratio and adverse clinical outcomes among LN patients. We demonstrated the potential predictive significance of the TC/HDL-C ratio by confirming significant correlations between the increased ratio at baseline and worse renal outcomes as well as all-cause mortality in LN. Our findings also provide compelling evidence linking the higher TC/HDL-C ratio to disease activity, as shown by significantly higher SLEDAI scores and lower serum C3 levels in LN patients exhibiting increased TC/HDL-C ratio.

Dyslipidemia has been reported to occur in approximately 50~70% of LN patients and is known to affect the long-term prognosis of LN. Dyslipidemia in LN often manifested as increased TC and decreased HDL-C 22, 23. Previous research has indicated that low HDL-C may indirectly promote tumor necrosis factor (TNF) -α release and the following inflammation in lupus status 6. We have previously investigated the connection between LN prognosis and HDL-C levels. However, we discovered that the effect of decreased HDL-C was not consistent on the adverse renal outcomes and all-cause death of LN, suggesting a limited predictive value of the single factor 9. The TC/HDL-C ratio, as a comprehensive lipid parameter, has been demonstrated to be correlated with the progression of various kidney diseases. Zhai et al. found that the increase of baseline TC/HDL-C was statistically related to the annual decline rate of eGFR in patients with normal eGFR 24. It has also been confirmed that among ESRD patients receiving peritoneal dialysis therapy, an elevated TC/HDL-C ratio was independently linked to overall mortality 25. The TC/HDL-C ratio was also discovered to be a distinct risk indicator for the advancement of CKD in a retrospective analysis involving 380 CKD patients 14. A non-linear relationship was discovered in a study utilizing data from the National Health and Nutrition Examination Surveys between the TC/HDL-C ratio and overall mortality, but not cardiovascular mortality in the normal population 12. However, the relationship between the TC/HDL-C ratio and LN progression still unknown.

Our findings were consistent with prior investigations and we found that the TC/HDL-C ratio levels were independently correlated to a higher risk of overall mortality and further revealed that the higher TC/HDL ratio was related to the incidence of adverse renal outcomes in LN. The adverse role of hyperlipidemia in chronic progressive kidney diseases was first proposed in 1982 26. The mechanistic association between hyperlipidemia and adverse renal outcomes may involve oxidative stress, endoplasmic reticulum stress, and inflammatory stress 27. Our observation of microthrombus in renal biopsies from patients with higher TC/HDL-C ratio was consistent with experimental evidence suggesting cholesterol enrichment in monocytes leading to the generation of pro-coagulant microvesicle and microthrombus formation 28, 29. In this study, we also found LN patients with increased TC/HDL-C ratio exhibited higher positive proportions of glomerular leukocyte infiltration. Consistent with our findings, previous study has demonstrated that hypercholesterolemia can result in pro-inflammatory response in glomerulus by producing chemotactic and adhesion molecules 30. These findings point to a potential role of hypercholesterolemia in renal pathological injury, a relationship that needs further investigation.

Subgroup analyses investigated whether the potential for prediction of the TC/HDL-C ratio altered by gender, age, hypertension, nephrotic syndrome, eGFR and pathological classification. The results revealed a particularly pronounced impact of TC/HDL-C ratio on poor clinical outcomes among male individuals, aged under 40 years or eGFR under 60 ml/min/1.73 m^2^ in LN. Multiple studies have elucidated that male and eGFR at flare are independent factors for adverse clinical outcomes in LN 31-35. That makes our study more significant, as it shows that the TC/HDL-C ratio has great predictive potential in those with high risks of adverse clinical outcomes in LN. Surprisingly, we also found that LN patients not manifested as nephrotic syndrome in the higher TC/HDL-C ratio group showed increased risks for adverse renal outcomes. Furthermore, LN patients with proliferative pathological changes in the higher TC/HDL-C ratio group showed significantly higher risk of all-cause mortality but no statistical significance for adverse renal outcomes, indicating that the prognostic value of the TC/HDL-C ratio may be more applicable to predicting mortality rather than renal outcomes in patients with proliferative LN. These results emphasize the necessity of considering these variables when applying the TC/HLD-C ratio to evaluate the prognosis of LN.

To our knowledge, this cohort study is the largest that has ever been carried out to investigate the relationship between the TC/HDL-C ratio and LN prognosis. Our findings extend previous work by demonstrating the consistent predictive value of the TC/HDL-C ratio for both adverse renal outcomes and all-cause mortality in LN. These findings broaden the application of lipid ratios and provide a feasible approach for the lipid management of LN patients. However, limitations also need to be noted. The potential impact of dynamic changes in maintenance therapy and complete data on cumulative dose or duration of glucocorticoids were not considered due to the unavailability of data on treatment changes during the long period of follow-up. Additionally, the therapies received before renal biopsy were not considered. The reliance on baseline lipid levels precludes the dynamic changes influenced by factors such as subsequent treatment or dietary habits. Also, given the limited number of the cardiovascular disease (CVD) mortalities, we did not investigate the connection between CVD mortality and the TC/HDL ratio in this cohort. To further validate our results and evaluate the impact of reducing the TC/HDL-C ratio on LN progression, more research is still required.

## Conclusion

The increased TC/HDL-C ratio was positively associated with poor prognosis in LN, providing evidence to support its application in predicting LN progression. Further studies of the TC/HDL-C ratio are necessary to confirm the optimal range and provide direction for the advancement of clinical lipid management in LN.

## Supplementary Material

Supplementary figure and tables.

## Figures and Tables

**Table 1:** Baseline characteristics of lupus nephritis patients according to the total cholesterol/high-density lipoprotein cholesterol (TC/HDL-C) ratio.

Variables	Total (n=818)	TC/HDL-C ratio		*P*
		< 6.71 (n = 574)	≥6.71 (n = 244)	
Gender-female, %	681 (83.25)	487 (84.84)	194 (79.51)	0.062
Age, years	27 (21, 36)	28 (21, 36)	27 (20, 36)	0.202
Weight, kg	52 (47, 59)	52 (46, 58)	54 (47, 60)	0.024*
Smoking, %	22 (2.69)	18 (3.14)	4 (1.64)	0.226
Drinking, %	9 (1.10)	8 (1.39)	1 (0.41)	0.385
Hypertension, %	277 (33.86)	177 (30.84)	100 (40.98)	<0.005*
Nephrotic syndrome, %	225 (27.51)	125 (21.78)	100 (40.98)	< 0.001*
LN duration, months	3 (1,12)	3 (1,12)	2.5 (1,11)	0.418
Hemoglobin, g/L	100.84 ± 22.78	103.06 ± 21.91	95.61 ± 23.96	< 0.001*
eGFR, ml/min/1.73 m^2^	89.1(50.1, 121.0)	101.81 (62.17, 124.67)	61.32 (35.62, 100.94)	< 0.001*
Total cholesterol, mmol/L	5.8 (4.6, 7.2)	5.4 (4.3, 6.7)	6.8 (5.5, 9.0)	< 0.001*
Triglyceride, mmol/L	2.1 (1.5, 2.9)	1.8 (1.3, 2.5)	3.0 (2.2, 4.0)	< 0.001*
HDL-C, mmol/L	1.1 (0.8, 1.4)	1.2 (0.9, 1.5)	0.8 (0.6, 0.9)	< 0.001*
LDL-C, mmol/L	3.5 (2.6, 4.5)	3.23 (2.4, 4.1)	4.2 (3.1, 5.4)	< 0.001*
Lipid-lowing treatment, %	260 (31.78)	157 (27.35)	103 (42.21)	< 0.001*
Serum albumin, g/L	26.0 (21.0, 32.0)	28.30 (23.00, 34.00)	21.00 (18.00, 26.00)	< 0.001*
Urine protein, g/24h	2.0 (0.9, 4.1)	1.7 (0.8, 3.4)	3.0 (1.3, 5.4)	< 0.001*
SLEDAI scores	16 (12, 19)	14 (11, 18)	16. (13, 20)	< 0.001*
Positive ANA, %	797 (97.43)	555 (96.69)	242 (99.18)	0.039*
Positive anti-dsDNA, %	678 (82.89)	468 (81.53)	210 (86.07)	0.115
Complement C3, g/L	0.44 (0.29, 0.62)	0.47 (0.31, 0.66)	0.37 (0.25, 0.54)	< 0.001*
Complement C4, g/L	0.10 (0.06, 0.18)	0.10 (0.06, 0.18)	0.10 (0.06, 0.17)	0.406
Serum immunoglobulin G, g/L	11.10 (7.13, 15.80)	11.50 (7.62, 16.40)	9.87 (5.97, 14.30)	< 0.001*
ESR, mm/h	41 (23, 44)	41 (21, 41)	41(30, 56)	< 0.001*
Pathological Classification, %				0.019*
non-proliferative LN	245 (29.95)	186 (32.40)	59 (24.18)	
proliferative LN	573 (70.05)	388 (67.60)	185 (75.82)	
Activity index	7 (5, 9)	6 (4, 9)	7 (5, 10)	< 0.001*
Chronicity index	3 (2, 4)	3 (2, 4)	3 (2, 4)	0.034*
Crescents, %	415 (50.73)	272 (47.39)	143 (58.61)	0.003*
Glomerular sclerosis, %	341 (41.69)	227 (39.55)	114 (46.72)	0.057
Glomerular leukocyte infiltration, %	563 (68.83)	370 (64.46)	193 (79.10)	< 0.001*
Interstitial fibrosis, %	341 (41.69)	225 (39.20)	116 (47.54)	0.027*
Tubular atrophy, %	465 (56.85)	314 (54.70)	151 (61.89)	0.058
Platinum loop, %	210 (25.67)	130 (22.65)	80 (32.79)	0.002*
Microthrombus, %	139 (16.99)	80 (13.94)	59 (24.18)	< 0.001*
Glucocorticoids, %	808 (98.78)	568 (98.95)	240 (98.36)	0.719
Immunosuppressants, %	444 (54.28)	317 (55.23)	127 (52.05)	0.404

Abbreviations: TC, total cholesterol; HDL-C, high-density lipoprotein cholesterol; LN, lupus nephritis; eGFR, estimated glomerular filtration rate; LDL-C, low-density lipoprotein cholesterol; SLEDAI, systemic lupus erythematosus disease activity index; ESR, erythrocyte sedimentation rate. * <6.71 vs. ≥6.71: *P* < 0.05.

**Table 2:** Multivariate Cox regression analysis of the TC/HDL-C ratio with prognosis of lupus nephritis.

	Model 1		Model 2		Model 3	
	HR (95% CI)	*P*	HR (95% CI)	*P*	HR (95% CI)	*P*
**All-cause mortality**						
TC/HDL-C ratio<6.71	Ref		Ref		Ref	
TC/HDL-C ratio ≥6.71	1.70 (1.20 ~ 2.41)	0.003	1.39 (0.95 ~ 2.04)	0.092	1.51 (1.03 ~ 2.23)	0.036
TC/HDL-C ratio (continuous)	1.07 (1.02 ~ 1.11)	0.002	1.07 (1.02 ~ 1.13)	0.006	1.07 (1.02 ~ 1.12)	0.010
**Adverse renal outcomes**						
TC/HDL-C ratio<6.71	Ref		Ref		Ref	
TC/HDL-C ratio ≥6.71	1.83 (1.28 ~ 2.63)	0.001	1.42 (0.96 ~ 2.12)	0.080	1.57 (1.05 ~ 2.36)	0.028
TC/HDL-C ratio (continuous)	1.07 (1.03 ~ 1.12)	< 0.001	1.05 (1.01 ~ 1.10)	0.047	1.06 (1.01 ~ 1.11)	0.015

Abbreviations: HR, Hazard Ratio; CI, Confidence Interval. Model 1: Crude; Model 2: Adjusted for gender, age, weight, glucocorticoids, immunosuppressants, lipid-lowing treatment; Model 3: Adjusted for Model 2 plus eGFR, nephrotic syndrome, IgG, ESR, activity index, and chronicity index

## Abbreviations

LN: lupus nephritis
TC: total cholesterol
HDL-C: high-density lipoprotein cholesterol
HR: hazard ratio
CI: confidence interval
SLE: systemic lupus erythematosus
ESRD: end-stage renal disease
TG: triglycerides
LDL-C: low-density lipoprotein cholesterol
CKD: chronic kidney disease
ESR: erythrocyte sedimentation rate
eGFR: estimated glomerular filtration rate
SLEDAI: Systemic Lupus Erythematosus Disease Activity Index
ROC: receiver operating characteristic
K-M: Kaplan-Meier
RCS: restricted cubic splines
TNF: tumor necrosis factor
CVD: cardiovascular disease
