# Supplementary material for: Associations of the Total Cholesterol/high-density Lipoprotein Cholesterol Ratio with Outcomes in Lupus Nephritis
Source: Int J Med Sci. 2025 Apr 28;22(10):2398–407. doi: 10.7150/ijms.106393 (PMC12080575; doi:10.7150/ijms.106393)
Supplement: Supplementary file 1 — Supplementary figure and tables. [file ijmsv22p2398s1.pdf]

Associations of the total cholesterol/high-density lipoprotein cholesterol ratio with outcomes in lupus nephritis

Xiaolei Shi[1, 2*], Yaoyao Tang[1, 2*], Wang Xiang[1, 2], Jianwen Yu[1, 2], Xin Wang[1, 2], Hongjian Ye[1, 2,] Zhong Zhong[1, 2], Haishan Wu[1, 2], Ruihan Tang [1, 2†], Xi Xia [1, 2†], Wei Chen[1, 2†]

*These authors have contributed equally to this work and share first authorship.

[1] Department of Nephrology, The First Affiliated Hospital of Sun Yat-Sen University, People's Republic of China

[2] NHC Key Laboratory of Clinical Nephrology (Sun Yat-Sen University） and Guangdong Provincial Key Laboratory of Nephrology, People's Republic of China

Corresponding Author:

Ruihan Tang (0000-0001-5250-6239), Department of Nephrology, The First Affiliated Hospital of Sun Yat-sen University, 58th, Zhongshan Road II, Guangzhou, 510080, China

Tel: +86 18998472466 E-mail: tangrh8@mail.sysu.edu.cn

Xi Xia (0000-0003-3146-5567), Department of Nephrology, The First Affiliated Hospital, Sun Yat-sen University, 58th, Zhongshan Road II, Guangzhou 510080, China

Tel: +86 18998472466 E-mail: xiax29@mail.sysu.edu.cn

Wei Chen (0000-0001-7194-8040), Department of Nephrology, The First Affiliated Hospital of Sun Yat-sen University, 58th, Zhongshan Road II, Guangzhou, 510080, China

Tel：+86 13924150966 Email: chenwei99@mail.sysu.edu.cn

Additional File

Table S1. The ROC curve analysis of the TC/HDL-C ratio, TC, TG, HDL-C and LDL-C for predicting all-cause mortality and adverse renal outcomes in patients with lupus nephritis.

	Test	optimal cut-off value	Sensitivity	Specificity	AUC
**All-cause mortality**	TC/HDL-C ratio	6.71	0.426	0.726	0.575
	TC	5.03	0.419	0.666	0.533
	TG	3.45	0.248	0.843	0.527
	HDL-C	1.19	0.798	0.398	0.608
	LDL-C	3.11	0.457	0.611	0.519
**Adverse renal outcomes**	TC/HDL-C ratio	6.12	0.554	0.641	0.601
	TC	7.75	0.294	0.817	0.545
	TG	2.75	0.470	0.730	0.557
	HDL-C	0.88	0.462	0.678	0.564
	LDL-C	3.19	0.655	0.432	0.504

Abbreviations: ROC curve, receiver operating characteristic curve; AUC, area under the curve; TC, total cholesterol; TG, Triglyceride; HDL-C, high-density lipoprotein cholesterol, LDL-C, low-density lipoprotein cholesterol.

Table S2. Causes of death for patients who died during follow-up periods

Cause of death	Total (n=818)	TC/HDL-C ratio		*P*
		< 6.71 (n = 574)	≥6.71 (n = 244)	
Renal failure	21 (2.57)	13 (2.26)	8 (3.28)	0.402
Infection	35 (4.28)	24 (4.18)	11 (4.51)	0.833
Cardiovascular or cerebrovascular events	26 (3.18)	17 (2.96)	9 (3.69)	0.570
Others(including tumor, gastrointestinal bleeding, and accident)	12 (1.47)	6 (1.05)	6 (2.46)	0.124
Unknown	35 (4.28)	14 (2.44)	21 (8.61)	<0.001*
Total death	129 (15.77)	74 (12.89)	55 (22.54)	<0.001*

* <6.71 vs. ≥6.71: $P < 0.05$.

***Figure S1.* Associations between the TC, HDL-C and LDL-C groups and clinical outcomes in LN.**

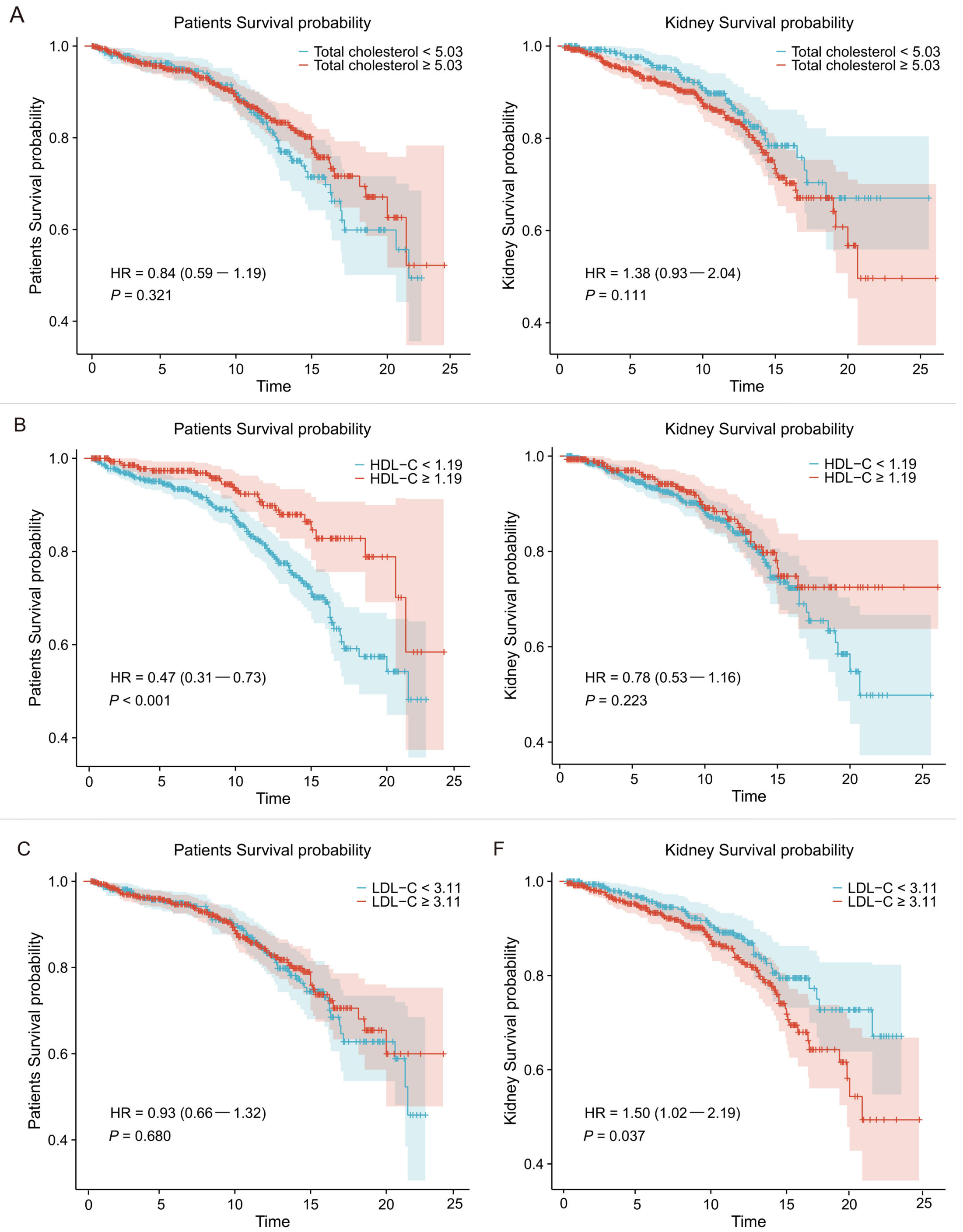

Kaplan-Meier survival curves for all-cause mortality and adverse renal outcomes of LN patients in the TC (A), HDL-C (B) and LDL-C (C) groups.
